# Increased isobutanol production in *Saccharomyces cerevisiae* by eliminating competing pathways and resolving cofactor imbalance

**DOI:** 10.1186/1475-2859-12-119

**Published:** 2013-12-05

**Authors:** Fumio Matsuda, Jun Ishii, Takashi Kondo, Kengo Ida, Hironori Tezuka, Akihiko Kondo

**Affiliations:** 1 Department of Bioinformatic Engineering, Graduate School of Information Science and Technology, Osaka University, 1-5 Yamadaoka, Suita, Osaka 565-0871, Japan; 2 Organization of Advanced Science and Technology, Kobe University, 1-1 Rokkodaicho, Nada, Kobe 657-8501, Japan; 3 RIKEN Center for Sustainable Resource Science, 1-7-22 Suehirocho, Turumi-ku, Yokohama, Kanagawa 230-0045, Japan; 4 Division of Natural Environment and Information, Faculty of Environment and Information Sciences, Yokohama National University, 79-7, Tokiwadai, Hodogaya, Yokohama 240-8501, Japan; 5 Department of Chemical Science and Engineering, Graduate School of Engineering, Kobe University, 1-1 Rokkodaicho, Nada, Kobe 657-8501, Japan

**Keywords:** Isobutanol, Ehrlich pathway, Single-gene deletion, Transhydrogenase-like shunt, *Saccharomyces cerevisiae*

## Abstract

**Background:**

Isobutanol is an important target for biorefinery research as a next-generation biofuel and a building block for commodity chemical production. Metabolically engineered microbial strains to produce isobutanol have been successfully developed by introducing the Ehrlich pathway into bacterial hosts. Isobutanol-producing baker’s yeast (*Saccharomyces cerevisiae*) strains have been developed following the strategy with respect to its advantageous characteristics for cost-effective isobutanol production. However, the isobutanol yields and titers attained by the developed strains need to be further improved through engineering of *S. cerevisiae* metabolism.

**Results:**

Two strategies including eliminating competing pathways and resolving the cofactor imbalance were applied to improve isobutanol production in *S. cerevisiae*. Isobutanol production levels were increased in strains lacking genes encoding members of the pyruvate dehydrogenase complex such as *LPD1*, indicating that the pyruvate supply for isobutanol biosynthesis is competing with acetyl-CoA biosynthesis in mitochondria. Isobutanol production was increased by overexpression of enzymes responsible for transhydrogenase-like shunts such as pyruvate carboxylase, malate dehydrogenase, and malic enzyme. The integration of a single gene deletion *lpd1*Δ and the activation of the transhydrogenase-like shunt further increased isobutanol levels. In a batch fermentation test at the 50-mL scale from 100 g/L glucose using the two integrated strains, the isobutanol titer reached 1.62 ± 0.11 g/L and 1.61 ± 0.03 g/L at 24 h after the start of fermentation, which corresponds to the yield at 0.016 ± 0.001 g/g glucose consumed and 0.016 ± 0.0003 g/g glucose consumed, respectively.

**Conclusions:**

These results demonstrate that downregulation of competing pathways and metabolic functions for resolving the cofactor imbalance are promising strategies to construct *S. cerevisiae* strains that effectively produce isobutanol.

## Background

There is increasing interest in the production of branched higher alcohols from renewable biomass to be used as a next-generation biofuel and as a building block for commodity chemical production [1,2]. Isobutanol is an important target for biorefinery research because of its preferable properties such as lower toxicity and higher octane values than its straight-chain counterpart [3]. Metabolically engineered microbial strains to produce isobutanol have been developed by introducing the Ehrlich pathway into bacterial hosts, including *Escherichia coli*, *Corynebacterium glutamicum*, *Clostridium cellulolyticum*, *Bacillus subtilis*, and cyanobacteria [4-14]. In the recombinant strains, 2-ketoisovalerate, which is an intermediate in the valine biosynthetic pathway, is converted into isobutanol in a two-step reaction: decarboxylation of 2-ketoisovalerate to isobutylaldehyde by 2-keto acid decarboxylase (KDC), and subsequent reduction to isobutanol by alcohol dehydrogenase (ADH) [4,15]. In bacterial hosts, isobutanol production near the theoretical maximal yield have been achieved by additional metabolic modifications such as deletion of competing pathways and resolving the cofactor imbalance caused by isobutanol production [7,8,13].

Baker’s yeast (*Saccharomyces cerevisiae*) has advantageous characteristics for cost-effective isobutanol production such as cell-recycling fermentation and tolerance against isobutanol and harsh conditions during fermentation [16]. Isobutanol-producing *S. cerevisiae* strains have been developed following a bacterial strategy by construction of the Ehrlich pathway in the cytosol through expression of the *kivd* gene from *Lactococcus lactis* and *ADH6* gene from *S. cerevisiae*[17]. Isobutanol production was increased by the additional activation of the innate valine biosynthetic pathway in mitochondria and by the overexpression of Ilv2p, Ilv5p, and Ilv3p in the cytosol to construct the artificial pathway [18-20]. It was recently reported that the construction of the Ehrlich pathway in mitochondria is effective to increase isobutanol production because of the compartmentalization of the isobutanol biosynthetic pathway [21]. However, the isobutanol yields and titers attained by the developed strains need to be further improved through engineering of *S. cerevisiae* metabolism. In this study, two strategies including eliminating competing pathways and resolving the cofactor imbalance were applied to improve isobutanol production in *S. cerevisiae*. Isobutanol production was increased by suppressing pyruvate dehydrogenase activity and by activating NADPH regeneration in the cytosol and mitochondria.

## Results

### Disruption of genes related to pyruvate metabolism and valine biosynthesis

**Figure 1:**
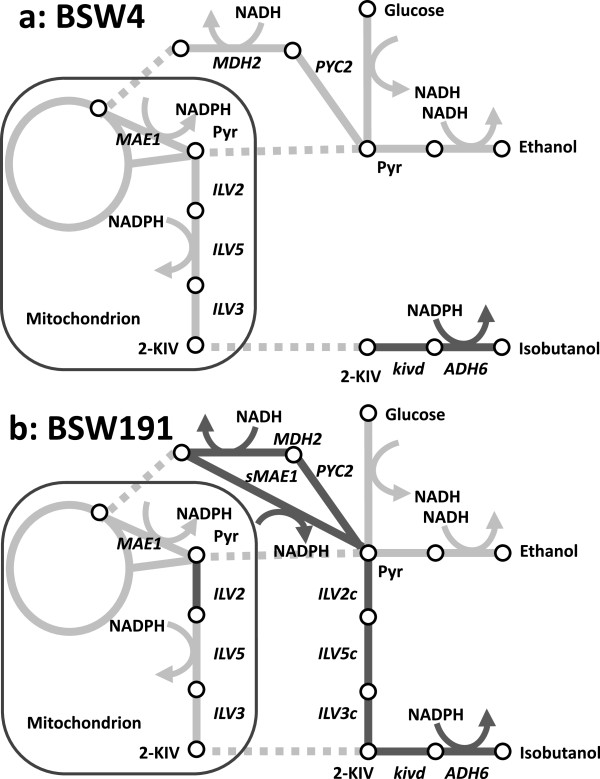
**Metabolic maps of the metabolically engineered *Saccharomyces cerevisiae *strains constructed in this study. (a)** BSW4 strain expressing two genes related to the Ehrlich pathway (*kivd* and *ADH6*). **(b)** BSW191 strain expressing genes related to the Ehrlich pathway (*kivd* and *ADH6*), mitochondrial valine biosynthetic pathway (*ILV2*), artificial valine biosynthetic pathway in the cytosol (*ILV2c, ILV5c,* and *ILV3c*), and transhydrogenase-like shunt (*PYC2, MDH2,* and *sMAE1*). Black lines indicate the overexpressed or constructed reactions in these strains. Dotted lines represent translocation between mitochondria and cytosol. Pyr, pyruvate; 2-KIV, 2-ketoisovalerate.

**Figure 2:**
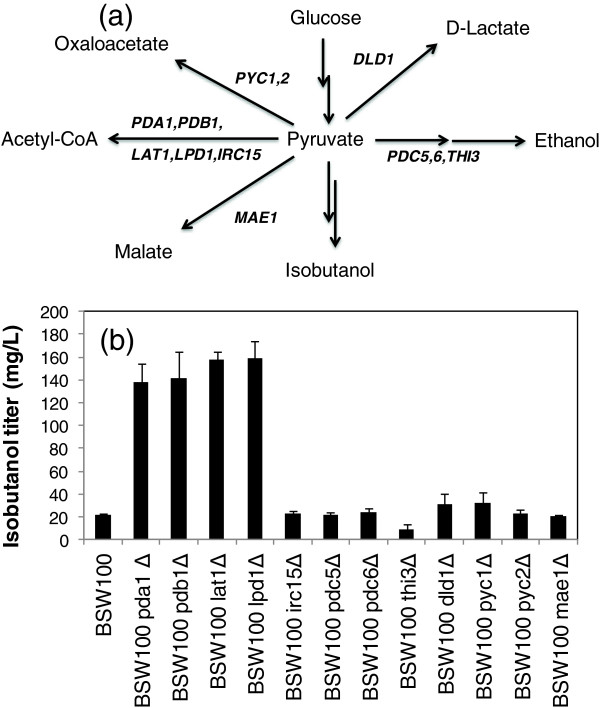
**Disruption of genes related to pyruvate metabolism. (a)** Genes investigated in this study. **(b)** Isobutanol production by single gene-deleted strains. All strains including the control strain (BSW100) were constructed by introducing pGK423-kivd, pGK425-ILV2, and pGK426-ADH6 plasmids. Isobutanol titers were determined at 72 h after the fermentation start. Each data point represents the mean (SD) values obtained from 3 replicate fermentations.

The metabolic map shown in Figure 1 indicates that pyruvate is a key intermediate in isobutanol biosynthesis because pyruvate is responsible for several metabolic functions such as the TCA cycle, anaplerotic pathways, and ethanol biosynthesis. Isobutanol production in *S. cerevisiae* was increased by knock-out of the *PDC1* gene [17], suggesting that disruption of other genes related to competing pathways should activate isobutanol biosynthesis. In this study, the effects of disruption of pyruvate metabolism-related genes on isobutanol production were examined by constructing single-gene knockout strains (Figure 2a). Three genes required for isobutanol biosynthesis, including *ILV2*, *kivd*, and *ADH6,* were introduced into 12 single-gene knockout strains. The constructed strains were cultivated in 5 mL of synthetic dextrose (SD) medium under semi-anaerobic conditions. Isobutanol concentration in the medium at 72 h after the start of cultivation was determined by using gas chromatography–mass spectrometry (GC-MS). The control strain (BSW100, Table 1) was constructed by introducing pGK423-kivd, pGK425-ILV2, and pGK426-ADH6 plasmids (Table 2) into the wild-type strain (BY4741) whose isobutanol production level was 22 ± 1 mg/L (Figure 2b).

**Table 1:** Yeast strains constructed in this study

**Strains**	**Genotypes**
YPH499	*MAT***a***ura3-52 lys2-801 ade2-101 trp1-*Δ*63 his3-*Δ*200 leu2-*Δ*1*
BY4741	*MAT***a***his3*Δ*1 leu2*Δ*0 met15*Δ*0 ura3*Δ*0*
BSW100	BY4741 /pGK423-kivd/pGK425-ILV2/pGK426-ADH6
BSW100 pda1Δ	BY4741 pda1Δ/pGK423-kivd/pGK425-ILV2/pGK426-ADH6
BSW100 pdb1Δ	BY4741 pdb1Δ/pGK423-kivd/pGK425-ILV2/pGK426-ADH6
BSW100 lat1Δ	BY4741 lat1Δ/pGK423-kivd/pGK425-ILV2/pGK426-ADH6
BSW100 lpd1Δ	BY4741 lpd1Δ/pGK423-kivd/pGK425-ILV2/pGK426-ADH6
BSW100 irc15Δ	BY4741 irc15Δ/pGK423-kivd/pGK425-ILV2/pGK426-ADH6
BSW100 pdc5Δ	BY4741 pdc5Δ/pGK423-kivd/pGK425-ILV2/pGK426-ADH6
BSW100 pdc6Δ	BY4741 pdc6Δ/pGK423-kivd/pGK425-ILV2/pGK426-ADH6
BSW100 thi3Δ	BY4741 thi3Δ/pGK423-kivd/pGK425-ILV2/pGK426-ADH6
BSW100 dld1Δ	BY4741 dld1Δ/pGK423-kivd/pGK425-ILV2/pGK426-ADH6
BSW100 pyc1Δ	BY4741 pyc1Δ/pGK423-kivd/pGK425-ILV2/pGK426-ADH6
BSW100 pyc2Δ	BY4741 pyc2Δ/pGK423-kivd/pGK425-ILV2/pGK426-ADH6
BSW100 mae1Δ	BY4741 mae1Δ/pGK423-kivd/pGK425-ILV2/pGK426-ADH6
BSW101	BY4741/pGK423/pGK425/pGK426
BSW101 pda1Δ	BY4741 pda1Δ/pGK423/pGK425/pGK426
BSW101 pdb1Δ	BY4741 pdb1Δ/pGK423/pGK425/pGK426
BSW101 lat1Δ	BY4741 lat1Δ/pGK423/pGK425/pGK426
BSW101 lpd1Δ	BY4741 lpd1Δ/pGK423/pGK425/pGK426
BSW4	YPH499/pATP426-kivd-ADH6/pATP423
BSW5	YPH499/pATP426-kivd-ADH6/pATP423-sMAE1
BSW6	YPH499/pATP426-kivd-ADH6/pATP423-MsM
BSW7	YPH499/pATP426-kivd-ADH6/pATP423-PMsM
BSW8	YPH499/pATP426-kivd-ADH6/pATP423-MAE1
BSW9	YPH499/pATP426-kivd-ADH6/pATP423-MM
BSW10	YPH499/pATP426-kivd-ADH6/pATP423-PMM
BSW13	YPH499/pATP426-kivd-ADH6/pATP425/pATP423
BSW14	YPH499/pATP426-kivd-ADH6/pILV2L/pATP423
BSW15	YPH499/pATP426-kivd-ADH6/pILV2L/pATP423-sMAE1
BSW16	YPH499/pATP426-kivd-ADH6/pILV2L/pATP423-MsM
BSW17	YPH499/pATP426-kivd-ADH6/pILV2L/pATP423-PMsM
BSW18	YPH499/pATP426-kivd-ADH6/pILV2L/pATP423-MAE1
BSW19	YPH499/pATP426-kivd-ADH6/pILV2L/pATP423-MM
BSW20	YPH499/pATP426-kivd-ADH6/pILV2L/pATP423-PMM
BSW187	BY4741/pATP426-kivd-ADH6-ILV2/pILV532cytM
BSW191	BY4741/pATP426-kivd-ADH6-ILV2/pILV532cytM/pATP423-PMsM
BSW192	BY4741/pATP426-kivd-ADH6-ILV2/pILV532cytM/pATP423-MAE1
BSW205	BY4741 lpd1Δ/pATP426-kivd-ADH6-ILV2/pILV532cytM/pATP423-MAE1
BSW206	BY4741 lpd1Δ/pATP426-kivd-ADH6-ILV2/pILV532cytM/pATP423-PMsM

**Table 2:** Plasmids used in this study

**Plasmid**	**Description**	**Source or reference**
pGK423	Yeast expression vector containing *PGK1* promoter, 2 *μ* origin, *HIS3* marker, no expression (control plasmid)	Ishii et al., 2009 [34]
pGK425	Yeast expression vector containing *PGK1* promoter, 2 *μ* origin, *LEU2* marker, no expression (control plasmid)	Ishii et al., 2009 [34]
pGK426	Yeast expression vector containing *PGK1* promoter, 2 *μ* origin, *URA3* marker, no expression (control plasmid)	Ishii et al., 2009 [34]
pATP423	Yeast three gene expression vector containing *ADH1*, *TDH3* and *PGK1* promoters, 2 *μ* origin, *HIS3* marker, no expression (control plasmid)	Ishii et al., in submission
pATP425	Yeast three gene expression vector containing *ADH1*, *TDH3*, and *PGK1* promoters, 2 *μ* origin, *LEU2* marker, no expression (control plasmid)	Ishii et al., in submission
pATP426	Yeast three gene expression vector containing *ADH1*, *TDH3*, and *PGK1* promoters, 2 *μ* origin, *URA3* marker, no expression (control plasmid)	Ishii et al., in submission
pGK423-kivd	pGK423, expression of *L. lactis* 2-ketoisovalerate decarboxylase (*kivd*) gene	Kondo et al., 2012 [17]
pGK425-ILV2	pGK425, expression of *S. cerevisiae ILV2* gene	Kondo et al., 2012 [17]
pGK426-ADH6	pGK426, expression of *S. cerevisiae ADH6* gene	Kondo et al., 2012 [17]
pILV532cytL	pATP425, co-expression of *S. cerevisiae ILV5c, ILV3c,* and *ILV2c* genes	This study
pILV532cytM	2 *μ* origin, *MET15* marker (pGK421-base), co-expression of *S. cerevisiae ILV5c, ILV3c,* and *ILV2c* genes	This study
pATP423-sMAE1	pATP423, expression of *S. cerevisiae sMAE1* gene	This study
pATP423-MsM	pATP423, co-expression of *S. cerevisiae sMAE1* and *MDH2* genes	This study
pATP423-PMsM	pATP423, co-expression of *S. cerevisiae sMAE1*, *MDH2, and PYC2* genes	This study
pATP423-MAE1	pATP423, expression of *S. cerevisiae MAE1* gene	This study
pATP423-MM	pATP423, co-expression of *S. cerevisiae MAE1* and *MDH2* genes	This study
pATP423-PMM	pATP423, co-expression of *S. cerevisiae MAE1*, *MDH2, and PYC2* genes	This study
pILV2L	pATP425, expression of *S. cerevisiae ILV2* gene	This study
pATP426-kivd-ADH6	pATP426, co-expression of *L. lactis kivd* and *S. cerevisiae ADH6* genes	This study
pATP426-kivd-ADH6-ILV2	pATP426, co-expression of *L. lactis kivd*, *S. cerevisiae ADH6,* and *ILV2* genes	This study

The fermentation results indicated that the five single-gene deleted strains, including BSW100 irc15Δ, BSW100 pdc5Δ, BSW100 pdc6Δ, BSW100 mae1Δ, and BSW100 pyc2Δ and had no positive or negative effects on isobutanol production. Among the genes tested, the BSW100 thi3Δ strain showed slightly reduced production of isobutanol (Figure 2b). Although this finding suggests that 2-ketoisovalerate may be a substrate of the decarboxylation reaction catalyzed by Thi3p, further characterization of Thi3p is essential for more detailed functional annotation. In contrast, isobutanol production levels were slightly increased in BSW100 dld1Δ and BSW100 pyc1Δstrains. Furthermore, isobutanol production was remarkably increased to 138–159 mg/L in BSW100 pda1Δ, BSW100 pdb1Δ, BSW100 lpd1Δ, and BSW100 lat1Δ strains (Figure 2b). The cell growth of these mutants was essentially same levels with that of wild type (data not shown). In order to estimate effects of these mutations alone on the isobutanol production, BSW101, BSW101 pda1Δ, BSW101 pdb1Δ, BSW101 lpd1Δ, and BSW101 lat1Δ strains were constructed by introducing the blank vectors (Table 1). The fermentation test demonstrated that isobutanol production levels were increased to 68–77 mg/L at 72 h after the fermentation start for the mutant strains (data not shown). Because the *PDA1, PDB1, LPD1,* and *LAT1* genes encode proteins in the pyruvate dehydrogenase complex, the results indicate that pyruvate supply for isobutanol biosynthesis is increased by reducing the activity of acetyl-CoA biosynthesis in the mitochondria.

### Implementation of transhydrogenase-like shunt

The metabolic pathway shown in Figure 1 also indicates that isobutanol biosynthesis requires NADPH as a cofactor for the reaction catalyzed by Ilv5p and Adh6p. For synthesis of one molecule of isobutanol from two molecules of pyruvate, reducing power has to be supplied by two molecules of NADPH. Because ethanol synthesis from pyruvate essentially uses NADH as a cofactor, the activation of isobutanol biosynthesis should cause NADPH shortage and NADH abundance. This cofactor imbalance could be relieved by the activity of pyridine nucleotide transhydrogenase catalyzing the following reaction: NADH + NADP^+^ → NAD^+^ + NADPH [22,23]. Although pyridine nucleotide transhydrogenase plays an important role in regulating the cellular redox state in many organisms, *S. cerevisiae* does not possess a gene encoding this enzyme [24]. Furthermore, heterologous expression of a bacterial transhydrogenase was not successful in *S. cerevisiae*[25].

Recently, it has been demonstrated that the metabolic shunt involving anaplerotic reactions functions similar to transhydrogenase [3,26,27]. Through this shunt, pyruvate is sequentially converted to oxaloacetate, malate, and pyruvate by the activity of pyruvate carboxylase (PYC), malate dehydrogenase (MDH), and malic enzyme (MAE), as shown in Figure 1. The net stoichiometry of the shunt is as follows: ATP + NADH + NADP^+^ → ADP + Pi + NAD^+^ + NADPH, because the coenzyme preferences of MDH and MAE in *S. cerevisiae* are NADH and NADP+, respectively [28]. The transhydrogenase-like shunt successfully resolved the cofactor imbalance in xylose-fermenting yeast expressing xylose reductase and xylulose dehydrogenase [27].

**Figure 3:**
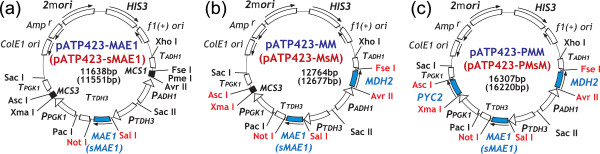
**Plasmids for co-expression of genes related to the transhydrogenase-like shunt. (a)** pATP423-MAE1 and pATP423-sMAE1 for the mitochondrial and cytosolic expressions of MAE1p. *sMAE1* is a short form of *MAE1* lacking the first 90 nucleotides. **(b)** pATP423-MM and pATP423-MsM for co-expressions of MDH2p and MAE1p. **(c)** pATP423-PMM and pATP423-PMsM for co-expressions of PYC2p, MDH2p and MAE1p*.*

In this study, two versions of transhydrogenase-like shuts were implemented to improve isobutanol production by distinct localization of malic enzyme (Mae1p), which is originally expressed in the mitochondria [29]. In the first version, Mae1p activity in the mitochondria was overexpressed to supply NADPH to the valine biosynthesis pathway. In the second version, NADPH was supplied to the Ehrlich pathway by the cytosolic expression of Mae1p (Figure 1b). For this, two plasmids, pATP423-MAE1 and pATP423-sMAE1, were constructed as shown in Figure 3a. The native *MAE1* gene and its truncated fragment (*sMAE1*) lacking the sequence of the mitochondrial transit signal [26] was inserted into an open reading frame (ORF) of the pATP423 plasmid. Plasmids additionally harboring *MDH2* and *PYC2* genes are also constructed by introducing the other ORFs of pATP423 plasmids (pATP423-MsM, pATP423-PMsM, pATP423-MM, and pATP423-PMM, Figure 3b and c). Mdh2p and Pyc2p are localized to the cytosol [27] (Figure 1).

**Figure 4:**
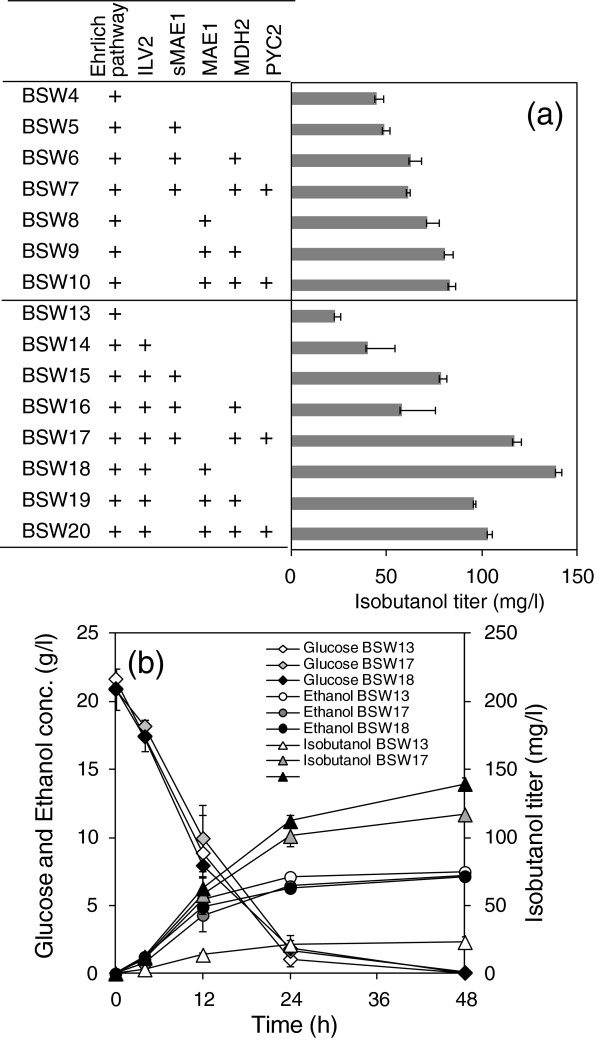
**Isobutanol production by transformants expressing genes related to the transhydrogenase-like shunt. (a)** Isobutanol production by *S. cerevisiae* strains co-expressing genes related to the Ehrlich pathway (*kivd* and *ADH6*), the *ILV2* gene, and transhydrogenase-like shunt. Isobutanol titers were determined at 48 h after the fermentation start. The introduced pathway and genes are shown in the figures. **(b)** Fermentation profiles of BSW13, BSW17 and BSW18. Detailed genotypes of each strain are described in Table 1. Each data point represents the mean (SD) values obtained from 3 replicate fermentations.

The pATP423-sMAE1, pATP423-MsM, and pATP423-PMsM plasmids were introduced into the YPH499 pATP426-kivd-ADH6 strain to construct a transhydrogenase-like shunt in the cytosol. There is a significant increase in isobutanol formation in BSW 6 and BSW 7 indicating that there is a slight activation of isobutanol production by implementation of the transhydrogenase-like shunt (Figure 4a). Furthermore, the BSW8 strain overexpressing mitochondrial MAE1p through introduction of the pATP423-MAE1 plasmid showed a 1.6-fold increase in isobutanol titer (71 ± 6 mg/L) at 48 h after the start of fermentation. Isobutanol production was activated by the additional overexpression of Mdh2p and Pyc2p in the BSW10 strain, and the isobutanol titer reached 83 ± 2 mg/L (Figure 4a).

The transhydrogenase-like shuts were also introduced for the strain whose isobutanol biosynthetic pathway in the mitochondria was activated by the overexpression of Ilv2p (Figure 4a). The isobutanol production in the control strain BSW13 (23 ± 3 mg/L, YPH499/pATP426-kivd-ADH6/pATP425/pATP423) is lower than that of BSW4 (45 ± 4 mg/L, YPH499/pATP426-kivd-ADH6/pATP423). Since the leucine biosynthesis is branched from the valine biosynthesis and thus competing with the isobutanol biosynthesis, the *leu2-*Δ*1*allele in BSW4 strain should have positive effect on isobutanol biosynthesis. On the other hands, the BMW13 strain showed a leucine-autotrophy by an additional introduction of ATP425 encoding *LEU2* gene. The isobutanol level was increased to 117 ± 6 mg/L by the overexpression of Mdh2p, Pyc2p, and cytosolic sMae1p (BSW17 strain, Figure 4a), probably because of the simultaneous upregulation of 2-ketoisovalerate and NADPH supply in cytosol for isobutanol synthesis via the Ehrlich pathway. Furthermore, activation of the NADPH supply in the mitochondria by overexpression of mitochondrial Mae1p also increased the isobutanol titer to 139 ± 4 mg/L, as shown for the BSW18 strain. The isobutanol yields of BSW17 and BSW18 strains were 0.006 ± 0.0003 and 0.007 ± 0.0002 g/g glucose consumed, respectively, since glucose was completely consumed at 48 h after the fermentation start (Figure 4b). The fermentation profiles indicated that the glucose consumption rates and the ethanol production rate of BSW17 and BSW18 were essentially identical with that of BSW13 (Figure 4b). The isobutanol titer was decreased, however, by additional introduction of the *MDH2* and *PYC2* genes (BSW19 and BSW20 strains), which suggests that there is an optimal balance of enzyme activities among pyruvate carboxylase, malate dehydrogenase, and malic enzyme.

### Construction of isobutanol overproducing strains

**Figure 5:**
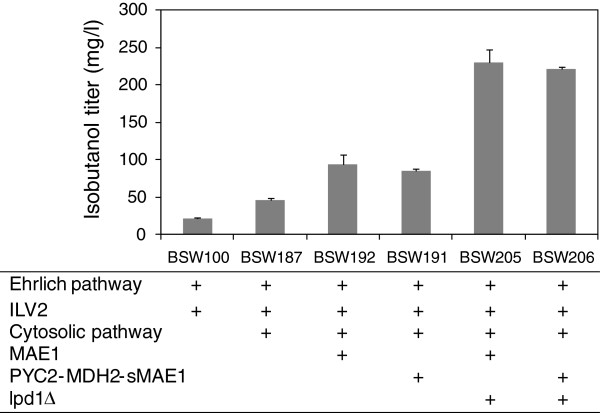
**Isobutanol production by metabolically engineered *S. cerevisiae *transformants.** These transformants are co-expressing genes related to the Ehrlich pathway (*kivd* and *ADH6*), activation of the mitochondrial valine biosynthetic pathway (*ILV2*), the artificial pathway for 2-ketoisovalerate biosynthesis in the cytosol (Cytosolic pathway) and two versions of transhydrogenase-like shunt (MAE1 and PYC2-MDH2-sMAE1) in combination with the single-gene deletion of *LPD1* (*lpd1*Δ). Isobutanol titers were determined at 48 h after the fermentation start. Each data point represents the mean (SD) values obtained from 3 replicate fermentations.

Metabolically engineered strains overproducing isobutanol were constructed by integrating the gene disruption and the transhydrogenase-like shunts examined in this study. Isobutanol production by the BSW187 strain (BY4741/pATP426-kivd-ADH6-ILV2/pILV532cytM) possessing genes for the Ehrlich pathway, activation of the mitochondrial valine biosynthetic pathway (ILV2), and the artificial pathway for 2-ketoisovalerate biosynthesis in the cytosol was 46 ± 14 mg/L (Figure 5). The cytosolic artificial pathway was constructed by the expression of truncated *ILV2c*, *ILV3c*, and *ILV5c* genes in which the mitochondrial translocation signals of the *ILV2*, *ILV3*, and *ILV5* genes were deleted. The details of the artificial pathway were described in our previous study [20]. It should be noted that the isobutanol production by the metabolically engineered strains with the BY4741 background were significantly lower than that by strains with the YPH499 background; the reason for this difference is unclear [17,20].

The isobutanol titer was increased by the additional introduction of transhydrogenase-like shunts as shown in BSW192 and BSW191 strains possessing pATP423-MAE1 and pATP423-PMsM plasmids, respectively. The isobutanol titer was increased to 94 ± 5 and 83 ± 2 mg/L by the activation of the NADPH supply in the cytosol and mitochondria, respectively. The cell growth was not affected by introducing pATP423-MAE1 and pATP423-PMsM plasmids (data not shown). The additional disruption of the *LPD1* gene in the BSW192 and BSW191 strains further activated isobutanol biosynthesis. The isobutanol titer of the BSW205 and BSW206 strains reached 230 ± 13 and 221 ± 27 mg/L, respectively (Figure 5) that correspond to isobutanol yields at 0.012 ± 0.0007 and 0.011 ± 0.001 g/g glucose consumed, respectively. The glucose were completely consumed at 48 h after the fermentation start (data not shown).

**Figure 6:**
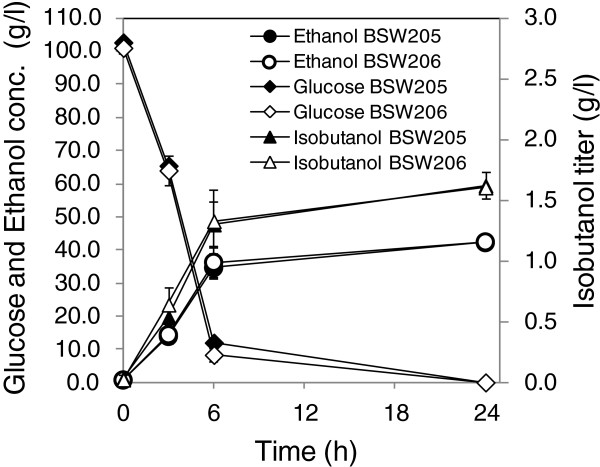
**Fermentation profiles of the BSW205 and BSW206 strains by batch fermentations at 50-mL scale under semi-anaerobic conditions.** The yeast cells were inoculated in 50 mL of SD medium containing 100 g/L glucose. Closed and open symbols represent data of BSW205 and BSW206, respectively. Circles, Diamonds, and Triangles represent the titers of ethanol, glucose, and isobutanol, respectively. Each data point represents the mean (SD) values obtained from 3 replicate fermentations.

The fermentation profile of the BSW205 and BSW206 strains was determined by batch fermentation at a 50-mL scale under semi-anaerobic conditions. The yeast cells were inoculated in 50 mL of SD medium containing 100 g/L glucose. As shown in Figure 6, the isobutanol titer reached 1.62 ± 0.11 and 1.61 ± 0.03 g/L at 24 h after the start of fermentation, which corresponded to isobutanol yields of 0.016 ± 0.001 g/g glucose consumed and 0.016 ± 0.0003 g/g glucose consumed, respectively. The fermentation test also demonstrated that the BSW205 and BSW206 strains actively consumed and produced glucose and ethanol, respectively. The ethanol yield of the BSW205 and BSW206 strains was 0.42 ± 0.01 and 0.42 ± 0.003 g/g glucose consumed, respectively.

## Discussion

In the metabolic engineering of microbial cell factories, elimination of competing pathways and resolution of the cofactor imbalance are essential to improve production of target compounds, as has been demonstrated in the construction of recombinant *E. coli* and *C. glutamicum* overproducing isobutanol [3,4,13]. In this study, these strategies were applied to increase isobutanol production in *S. cerevisiae*. The metabolic simulation of single gene-deletion strains demonstrated that suppression of ethanol biosynthesis by the deletion of alcohol dehydrogenase genes is effective to increase isobutanol yield [28]. Although a recombinant *S. cerevisiae* strain lacking five alcohol dehydrogenase genes has recently been constructed [30], this strategy is unlikely to be effective because the Ehrlich pathway for isobutanol biosynthesis also requires an alcohol dehydrogenase gene (*ADH6*). Thus, to find other gene deletion targets, the isobutanol production of 12 single gene-deletion mutants expressing genes for isobutanol biosynthesis was experimentally investigated (Figure 2). A fermentation test showed that isobutanol production was significantly increased in strains lacking genes responsible for the pyruvate dehydrogenase (PDH) complex. In these strains, PDH activity was reduced but not eliminated by the deletion of a single protein of the complex, which resulted in increased carbon flux into isobutanol biosynthesis. These results highlight mechanisms regulating isobutanol biosynthesis in *S. cerevisiae* that could not be identified by computer simulation of metabolism based on metabolic flux balance analysis.

In the second part of this study, the cofactor imbalance in isobutanol-producing *S. cerevisiae* strains was resolved by introducing transhydrogenase-like shunts (Figure 4 and 5). The transhydrogenase-like shunt consisted of Pyc2p, Mdh2p, and Mae1p originally existing in *S. cerevisiae* (Figure 1) [26,31]. However, the shunt was hardly functional in *S. cerevisiae* because the deletion of these genes showed no negative effect on isobutanol production (Figure 2). The recombinant strains overexpressing Mae1p showed improved production of isobutanol (Figure 4a), which indicates that increased NADPH and pyruvate supply in mitochondria through the activation of mitochondrial malic enzyme (Mae1p) could be a driving force to increase isobutanol biosynthesis. It was also demonstrated that isobutanol production in *S. cerevisae* was increased by the expression of malic enzyme in the cytosol (sMae1p) to supply NADPH in the cytosol (Figure 4a). The effect of the transhydrogenase-like function on NADH/NAD^+^ and NADPH/NADP^+^ levels was confirmed in xylose-fermenting *S. cerevisae* overexpressing the shunt [27]. The improvement of isobutanol production in *S. cerevisiae* by resolving the cofactor imbalance was demonstrated for the first time in this study.

The integration of PDH suppression by *lpd1*Δ and activation of the transhydrogenase-like shunt in BSW205 and BSW206 strains successfully increased the isobutanol levels to 230 ± 13 and 221 ± 27 mg/L, respectively (Figure 5). In the batch fermentation test at the 50-mL scale from 100 g/L glucose using these recombinant strains, the isobutanol titer reached 1.62 ± 0.11 and 1.61 ± 0.03 g/L at 24 h after the start of fermentation (Figure 6). The titer corresponds to the yield at 0.016 ± 0.001 and 0.016 ± 0.0003 g/g glucose consumed, respectively.

## Conclusions

All recombinant strains constructed in this study chiefly produced ethanol. For instance, the ethanol yield from glucose of BSW205 and BSW206 strains in the batch fermentation test at the 50-mL scale was 0.42 ± 0.01 and 0.42 ± 0.003 g/g glucose consumed, respectively (Figure 6), which indicates that restriction of ethanol biosynthesis from pyruvate is unavoidable to drastically improve isobutanol yield. However, it has been demonstrated that downregulation of ethanol biosynthesis by deleting the pyruvate decarboxylase and alcohol dehydrogenase genes seriously hampers active cell growth [30,32]. In this regard, identification of the *MTH1ΔT* allele is a promising because the mutation enables *S. cerevisiae* strains lacking three pyruvate dehydrogenase genes to actively grow with reduced ethanol production [33]. Recombinant *S. cerevisiae* strains effectively producing isobutanol will be constructed by integrating further activation of pathways for isobutanol biosynthesis [19-21], the down-regulation of competing pathways for acetyl-CoA and ethanol synthesis, and metabolic functions for resolving the cofactor imbalance as demonstrated in this study.

## Methods

### Strains, plasmids, and yeast transformation

The yeast strains used in this study are listed in Table 1. *S. cerevisiae* YPH499 (*MAT***a***ura3-52 lys2-801 ade2-101 trp1-*Δ*63 his3-*Δ*200 leu2-Δ1*, purchased from Stratagene, La Jolla, CA, USA) (Sikorski and Hieter, 1989), BY4741 (*MAT***a***his3*Δ*1 leu2*Δ*0 met15*Δ*0 ura3*Δ*0*) and the single gene deletion mutants (purchased from Thermo Scientific) were used for yeast host strains. The plasmids and primers used in this study are summarized in Tables 2 and 3, respectively. All plasmids were derived from the pGK and pATP vector series, in which gene expression is controlled by the *PGK1* promoter and the *ADH1*, *TDH1*, and *PGK1* promoters, respectively [34] (Ishii et al., in submission). The *kivd* gene from *Lactococcus lactis* was synthesized by Invitrogen Life Technologies Corp. (Carlsbad, CA). All other genes derived from *S. cerevisiae* were amplified from YPH499 genomic DNA using primers shown in Table 3. A short form of *MAE1* lacking the first 90 nucleotides (*sMAE1*) was cloned from the YPH499 genome using sMAE1 (fw) and sMAE1 (rv) primers. pILV532cytM was constructed by inserting a *Xho*I/*Sac*I-digested fragment of pILV532cytL into the same sites of the pGK421 vector harboring the *MET15* marker and a 2 *μ* origin backbone. The growth conditions, DNA techniques, and lithium-acetate method for transformations were previously described [35,36].

**Table 3:** Primers used in this study

**Target genes**	**Primers**	**Restriction enzymes**
*ILV5c (fw)*	5′-ttttCCTAGGatgttgaagcaaatcaacttcggtggtact	*Avr*II
*ILV5c (rv)*	5′-ttttGGCCGGCCttattggttttctggtctcaactttctg	*Fse*I
*ILV3c (fw)*	5′-aaaaGTCGACatgctttatgccaccggtttcaagaaggaa	*Sal*I
*ILV3c (rv)*	5′-ggggGCGGCCGCtcaagcatctaaaacacaaccgttggaa	*Not*I
*ILV2c (fw)*	5′-ccccCCCGGGatgccagagcctgctccaagtttcaatgtt	*Xma*I
*ILV2c (rv)*	5′-ttttGGCGCGCCtcagtgcttaccgcctgtacgcttatga	*Asc*I
*MDH2 (fw)*	5′-aaaaCCTAGGatgcctcactcagttacacc	*Avr*II
*MDH2 (rv)*	5′-aaaaGGCCGGCCttaagatgatgcagatctcg	*Fse*I
*sMAE1 (fw)*	5′-ttttGTCGACatgtggcctattcagcaatcgcg	*Sal*I
*sMAE1 (rv)*	5′-ttttGCGGCCGCctacaattggttggtgtgca	*Not*I
*MAE1 (fw)*	5′-ccccGTCGACatgcttagaaccagactatc	*Sal*I
*MAE1 (rv)*	5′ttttGCGGCCGCctacaattggttggtgtgca	*Not*I
*PYC2 (fw)*	5′-aaaaCCCGGGatgagcagtagcaagaaatt	*Xma*I
*PYC2 (rv)*	5′-aaaaGGCGCGCCttactttttttgggatgggg	Asc I
*ADH6 (fw)*	5′ggggCCTAGGatgtcttatcctgagaaa	*Avr*II
*ADH6 (rv)*	5′-aaaaGGCCGGCCctagtctgaaaattctttgt	*Fse*I
*kivd (fw)*	5′-ccccGTCGACatgtatacagtaggagatta	*Sal*I
*kivd (rv)*	5′-ccccGCGGCCGCttatgatttattttgttcag	*Not*I

### Isobutanol fermentation

The transformants were cultured for 72 h at 30°C in 5 mL of SD minimal medium (6.7 g/L yeast nitrogen base without amino acids and 20 g/L glucose) containing the required amino acids. Following centrifugation at 3,000 rpm for 5 min and removal of supernatant, the yeast cells were cultured in 5 mL of fresh SD minimal medium containing the required amino acids. The concentrations of isobutanol and ethanol in the medium at 48 and 72 h after the start of fermentation were determined using GC-MS (GCMS-QP2010 Plus; Shimadzu) following a previously described procedure [17]. The glucose concentrations were determined by Glucose C-II Test Wako Kit (Wako Pure Chemicals, Tokyo, Japan).

For flask-scale fermentation, the yeast transformants were aerobically cultivated in SD minimal medium containing the required amino acids for 48 h at 30°C. The cells were collected by centrifugation at 1,000 × *g* for 5 min at 4°C and washed twice with sterile water. The cells were then inoculated into 50 mL of YP medium (containing 10 g/L yeast extract and 20 g/L peptone) with 100 g/L glucose. The initial cell concentration was adjusted to 30 g of wet cells per liter (corresponding to OD 20 and 6.7 g of dry cells per liter). All fermentations were performed at 30°C with mild agitation in 100-mL closed bottles equipped with a bubbling CO_2_ outlet.

## Competing interests

We declare that the authors have no conflicts of interest in connection with this paper.

## Authors’ contributions

JI, TK, FM, HT, and KI performed the experiments. FM, JI, and TK analyzed the data. FM, JI, TK, and AK designed the study. JI, FM, and JI wrote the paper. All authors read and approved the final manuscript.
